# Intestinal Peristalsis

**Published:** 1902-01-04

**Authors:** 


### INTESTINAL PERISTALSIS.

By the use of the X-rays it seems possible that many points in regard to the physiology of the internal organs may be cleared up, although at present, with full knowledge of the uncertainty which so often occurs in correctly interpreting the appearances on the screen, we may well hesitate to accept without reserve some of the suggestions which have lately been put forward. The results of recent observations in regard to intestinal peristalsis are, however, very interesting. Dr. Walter B. Cannon has lately described what he saw when investigating the intestinal digestion of the cat by aid of the X-rays. By mixing the food with bismuth, which markedly obstructs the passage of the rays, and thus produces a well-marked shadow, it was found possible to watch the effects of the intestinal movements upon their contents. We have been in the habit of regarding the action of the intestinal muscles as taking the form of a wave-like contraction sweeping onwards in such a manner as to drive the contents forward. Starling[1] describes it thus:—"The movements of the intestines are typically peristaltic, and consist of an annular wave of constriction passing along the whole length of the intestine from above downwards." What Dr. Cannon appears to have seen, however, is something very different. He first sees the bismuth-stained chyme lying in a loop of intestine; then after a momentary agitation he sees the mass divided by a series of constrictions which appearing at intervals along the food mass appear to cut it into little bits. In a couple of seconds each of these segments becomes divided again, the former constriction passing away and allowing the divided food mass to be rejoined where it had been separated. Thus instead of the older view of the muscular contractions sweeping the chyme forward, we get an idea of the intestines grasping their contents, dividing and subdividing them, squeezing them and letting them go, and thus not only churning up the chyme but possibly aiding its entry into the capillaries and lacteals by the positive pressure produced by the contraction of the intestinal muscles. If this should turn out to be a correct conception of what goes on during digestion, loss of muscular tone would assume a new importance in the production of dyspepsia. We all admit the importance of muscular action in securing a regular action of the bowels, and the advantages derived from the administration of nux vomica and the use of massage in overcoming chronic constipation, and we all see, every day of our lives, that when constipation is thus overcome digestion improves and many other troubles are relieved; but it is probably by no means fully recognised that digestion and absorption are themselves so largely the result of muscular contractivity as would seem to be the case if Dr. Cannon has rightly interpreted the indications given by the X-rays, and that improved muscular activity may as likely as not have its first effect upon digestion and only secondarily upon the excretion of the residuum.

[1] Elements of Human Physiology.

